# A Pilot Study into the Association between Oral Health Status and Human Papillomavirus—16 Infection

**DOI:** 10.3390/diagnostics7010011

**Published:** 2017-03-01

**Authors:** Charles Xiaohang Sun, Nigel Bennett, Peter Tran, Kai Dun Tang, Yenkai Lim, Ian Frazer, Lakshman Samaranayake, Chamindie Punyadeera

**Affiliations:** 1 Institute of Health and Biomedical Innovation, The School of Biomedical Sciences, Queensland University of Technology, 60 Musk Avenue, Kelvin Grove, GPO Box 2434, Brisbane, QLD 4059, Australia; xiaohang.sun@uqconnect.edu.au (C.X.S.); n.bennett@qut.edu.au (N.B.); peter.tran1@uqconnect.edu.au (P.T.); kai.tang@qut.edu.au (K.D.T.); y43.lim@qut.edu.au (Y.L.); 2 School of Dentistry, Faculty of Health and Behavioural Sciences, The University of Queensland, 288 Herston Road, Herston, Brisbane, QLD 4006, Australia; l.samaranayake@uq.edu.au; 3 School of Medicine, Faculty of Medicine and Biomedical Sciences, The University of Queensland, 288 Herston Road, Herston, Brisbane, QLD 4006, Australia; i.frazer@uq.edu.au

**Keywords:** human papillomavirus (HPV), oropharyngeal cancer, oral cancer, head and neck cancer, saliva diagnostics, oral rinse, oral health, periodontal health, smoking, alcohol

## Abstract

Background: Over the next 20 years, oropharyngeal cancers (OPC) will represent the majority of head and neck cancers (HNCs) in the United States. It is estimated that human papillomavirus (HPV) may account for as much as 70% to 80% of OPCs in North America and in certain parts of Europe. It is hence crucial to understand the disease risk factors and natural history of oral HPV infections. We hypothesized that poor oral health (by measures such as poor oral hygiene and periodontal disease) leads to a higher degree of oral HPV-16 infections within a patient cohort from a dental school clinic. This study aims to test this hypothesis and gauge possible disease associations before larger scale studies. Subjects and Methods: 223 participants were recruited in this study from the University of Queensland Dental School clinic. Clinical oral health parameters (such as oral hygiene measures and periodontal disease measurements) have been examined and determined by dental professionals. We have collected oral rinse samples from these volunteers. Results: 10 (4.5%) out of 223 participants were found to have HPV-16 DNA in their oral rinse samples using NB2 endpoint PCR and Sanger sequencing. Within the HPV-16 DNA positive subjects, 7 (70%) and 3 (30%) were associated with poor oral hygiene and periodontal disease, respectively. Conclusion: Our results show a trend towards a positive correlation between oral HPV-16 infection and poor clinical oral health status.

## 1. Introduction

Over the next 20 years, oropharyngeal cancers (OPC) will represent the majority of head and neck cancers (HNCs) in the United States [1], while oral cavity cancers (OC, mainly non-human papillomavirus (HPV)-16) are increasing in emerging economies [2]. More than half of OPC and OC tumours are diagnosed at an advanced stage [3], and only 50% of OC patients and 66% of OPC patients survive beyond 5 years (American Cancer Society, 2015). Furthermore, 30% of OPC patients develop recurrences and metastases within 2 years [2]. OPC is generally caused by oral or oropharyngeal infection with HPV and, at any given time, approximately 7% of the population will have an oral or oropharyngeal HPV infection [4,5]. The lifetime oral HPV exposure rate is unknown, but an estimated 65%–100% of sexually active adults have been exposed to HPV at any anatomic site (oral, genital, or anal) [6,7,8]. Men are more likely to have an oral HPV infection than women [4]. It is estimated that HPV may account for as much as 70% to 80% of OPCs in North America and in certain parts of Europe [8,9,10].

HPV is a double-stranded DNA virus, infects basal cells of the epithelium lining and has been documented to cause various epithelial lesions. More than 30 genotypes of mucosotropic HPV have been identified with low-risk types (most common 6, 11) being associated with benign lesions and high-risk types (most common 16, 18) being associated with malignant conditions (such as cervical malignancy and OPC) [11,12,13]. HPV-16-driven OPC constitute 90% of HPV-positive OPC [14]. The aetiology and risk factors of oral and oropharyngeal HPV infection remains controversial; however, past studies have shown that these risk factors include oral sex, multiple sexual partners, poor oral hygiene and immunodeficiency conditions [15,16,17,18,19,20]. Identifying key risk factors may help to improve disease screening processes to identify high-risk individuals for developing OPC which may lead to OPC prevention and improve disease mortality [21].

The oral cavity and oropharynx are lined with various types of specialised epithelia (i.e., mucosal squamous epithelium) [22]. Due to HPV’s known basal cell tropism, it is logical to examine the epithelial components within these regions when studying HPV infections [22]. Previous studies have shown that self-reported poor oral hygiene is an independent risk factor for high-risk HPV infections [15,23]. The most common pathology involving oral epithelium is gingivitis—the inflammation of gums. Gingivitis is a non-specific and reversible gingival inflammation in response to oral plaque biofilms [24]. Untreated gingivitis in susceptible patients may progress to periodontitis (the inflammation which features irreversible damage to the gingiva and supporting tissues of the tooth, periodontium) of which the most common type is chronic periodontitis [24].

We hypothesised that poor clinical oral health (measures such as oral hygiene and periodontal disease) leads to a higher degree of oral HPV-16 infections within this dental school patient population. To test this hypothesis, we recruited participants (*n* = 223) with both good and bad oral hygiene and determined their periodontal disease status, as well as other oral health parameters. This is the first study to report clinical oral health parameters examined and evaluated by dental professionals and not self-reported oral health status. We found that oral HPV-16 infection within the Australian Dental study group is 4.5%. Our results demonstrated a positive correlation between oral HPV-16 infection and poor oral health status.

## 2. Methods

### 2.1. Participants

A total of 223 patients attending The University of Queensland School of Dentistry (UQDS) Clinic between October 2014 and October 2015 were recruited as study participants (see Table 1). All participants provided informed consent prior to the study which was approved by the UQ medical research ethics committee (Approval number 2014000862, 22 July 2014), and Queensland University of Technology (QUT) university human research ethics committee (Approval number 1400000641, 11 September 2014). Both approvals were obtained in 2014. The study was performed in accordance with the Declaration of Helsinki.

Participant inclusion criteria included (1) willingness to participate and provide written informed consent; (2) never received HPV vaccination; (3) older than 18 years of age (to exclude disparities due to sexual transmission of HPV) [15,25,26,27]; (4) no history of cancer and not receiving chemotherapy or radiation therapy, (malignancies and their treatment profoundly affect oral health status and immune status, which may increase HPV infection rates compared to general population rates) [15,25,26,27,28]; (5) no history of diabetes mellitus, cardiovascular diseases, blood disorders, xerostomia and any other systemic conditions that may affect oral health status) [15,25,26,27,29]; (6) not on current medication; (7) at least 20 teeth in the mouth, to evaluate the effect of periodontal infection on oral HPV infection.

### 2.2. Participant Demographics, Lifestyle and Clinical Data

Participant demographic information included in this study was collected for gender, age and race (see Supplementary Tables S1 and S2). Participant lifestyle factors such as smoking and alcohol consumption status were recorded. Participants’ race was classified as Caucasian, Asian, South Asian, Aborigine or Pacific Islander, African, Latino and other ethnicity groups. Participant smoking status was recorded based on the Centre of Disease Control smoking classification as follows: never smoked; former smoker; current daily smoker (1–15 cigarettes/day); current daily smoker (25–34 cigarettes/day); current daily smoker (>35 cigarettes/day); and current non-daily smoker (<1 cigarettes/day) [30]. Participant alcohol consumption status was recorded based on the U.S. Centre for Nutrition Policy and Promotions—Dietary Guidelines for Americans as follows: non-drinker, infrequent drinker (<1 standard drinks/week), regular drinker (<1 standard drink per day, female; <2 standard drinks per day, male), regular drinker (>1 standard drink per day, female; >2 standard drinks per day, male) [31] All participant demographic and lifestyle measures were confirmed with the participant verbally and recorded at the time of collection.

Participant clinical oral health measures were recorded, including plaque level, supra-gingival calculus level, sub-gingival calculus level (all three parameters were graded as mild—detected on less than 30% of the total number of teeth, moderate—detected on 30% to 60% of the total number of teeth, and severe—detected on more than 60% of the total number of teeth), periodontal screening record (PSR) (score 0–4, for each of six divided anatomical sextants in the mouth: score 0 = no bleeding on probing (BOP) and calculus present, no periodontal pocket probing depth greater than 3.5 mm; score 1 = BOP present, no calculus present, no periodontal pocket probing depth greater than 3.5 mm; score 2 = BOP and calculus present, no periodontal pocket probing depth greater than 3.5 mm; score 3 = BOP and calculus present, periodontal pocket probing depths greater than 3.5 mm but less than 5.5 mm, score 4 = BOP and calculus present, periodontal pocket probing depths greater than 5.5 mm) [32] and decayed, missing and filled teeth (DMFT) number (0–28). All oral health measurements were extracted from participants’ most recent oral examination records at the UQDS, at the time of sample collection; the measurement scores were determined by an operating training dentist who conducted the oral examination and the findings were verified by a qualified supervising dentist.

Participants were grouped based on their periodontal disease status. If any of the six PSR scores were greater than 3, the patient was classified as a periodontal-diseased patient [32]. If none of the six PSR scores were greater than 2, the patient was classified as a non-periodontal-diseased patient [32]. Participants were also grouped based on their oral hygiene status. If any of the plaque and supra/sub-gingival calculus scores were greater than the moderate level, the participant was classified as having poor to fair oral hygiene (as opposed to good to excellent oral hygiene).

### 2.3. Salivary Oral Rinse Sample Collection and Processing

Appropriate environmental controls and blinded blank saline samples were collected at the UQDS to ensure that the sample are not contaminated by HPV-16 virus aerosols. Prior to sample collection, participants were asked to rinse their mouths with water to remove any food debris and other irrelevant material. Participants were then instructed to use 20 mL of 0.9% saline to swish and gargle alternatively for 30 s (alternate every 5 s) and spit the sample into a sterile collection tube as per our previous work [16,33]. The samples were then transported on dry ice and stored at −80 °C at QUT adhering to local storage procedures and protocols, until further processed. Clinical and laboratory staff were double-blinded through a process of sample de-identification in order to reduce the risk of bias.

### 2.4. DNA Extraction from Oral Rinse Samples

**Figure 1:**
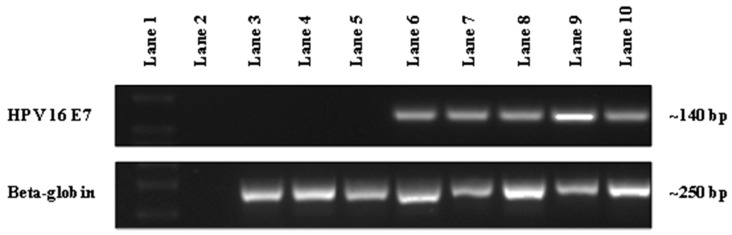
The detection of human papillomavirus (HPV)-16 DNA in patient oral rinse samples from UQ Dental School Clinic by PCR. Representative gel image showing the detection of HPV-16 DNA in patient oral rinse samples using HPV-16 NB2 primers (~140 bp) with beta-globin used as an internal control (~250 bp). Lane 1 represents DNA Ladder; Lane 2 represents non-template control; Lanes 3–5 (134, 144 and 145) represent the absence of HPV-16 DNA in patient samples; Lanes 6–8 (236, 1338 and 1368) represent the presence of HPV-16 DNA in patient samples and Lane 9 (93VU147T) and 10 (Caski) represent HPV-16 positive cancer cell lines.

Oral exfoliated cell pellets were resuspended in sterile phosphate-buffered saline (PBS) and DNA was extracted using the QIAmp DNA Mini Kit, Catalog number 51304 (Qiagen, Hilden, Germany) according to the manufacturer’s instructions. DNA samples were assessed for purity and quantified on a Nanodrop 1000 Spectrophotometer (Thermo Fisher Scientific, Pittsburgh, PA, USA) (see Figure 1).

### 2.5. End-Point PCR Detection and Sequence Confirmation of HPV-16 DNA

Two rounds of end-point PCR were used to detect HPV-16 DNA extracted from oral rinse samples. HPV-16 specific primers were used to amplify a region of the E7 gene. Human specific primers were used to detect β-globin as a reference gene to normalise for DNA input and integrity. HPV-16 primer sequences; NB2 Forward primer 5′-AGTGTGACTCTACGCTTCGG-3′, NB2 Reverse primer 5′-CTGCAGGATCAGCCATGGTA-3′. β-globin primers sequences; Forward primer 5′-CAACTTCATCCACGTTCACC-3′, Reverse primer 5′-GAAGAGCCAAGGACAGGTAC-3′. The specificity of NB2 primers was ascertained by isolating DNA from the HPV-positive cell line (SCC-2) and amplifying, followed by Sanger sequenceing identification (see Figure 1 and Supplemental Figure S1).

The PCR setup consisted of 50 ng of DNA isolated from oral rinse samples, 1 µM of each primer, 1× Emerald AMP MAX HS PCR Mastermix (Takara Bio, Otsu, Shiga, Japan) in a final volume of 12.5 µL. PCR cycling consisted of an initial denaturation at 95 °C for 3 min followed by 40 cycles of denaturation at 95 °C for 30 s; annealing at 65 °C for 30 s for HPV-16 NB2 primers, or 60 °C for β-Globin primers; and extension at 72 °C for 30 s. A final extension at 72 °C for 5 min before cooling to 12 °C was done. A 1 µL volume was transferred to another PCR setup tube and the above reaction conditions were repeated. The entire volume from the second post-PCR samples were subjected to gel electrophoresis at 120 V for 50 min in a 3% agarose gel prepared in TAE buffer (Tris base, acetic acid and EDTA).

PCR amplicons of correct size were excised and gel purified using the QIAquick Gel Extraction Kit, Catalog number 28704 (Qiagen, Hilden, Germany) according to the manufacturer’s instructions. Gel purified NB2 amplicons were used in single primer PCR’s (NB2 reverse primer) with BigDye^®^ Terminator v3.1 Cycle Sequencing Kit Catalog number: 4337458 (Thermo Fisher Scientific, Waltham, MA, USA). Sanger sequencing clean-up and separation was performed by the Australian Equine Genetics Research Centre, University of Queensland. Specifically, BigDye sequencing reactions were cleaned up with the Agencourt CleanSEQ System (Beckman-Coulter, Brea, CA, USA). Capillary electrophoresis and fluorescence detection of cleaned-up reactions were done on either a 3130XL or 3730 Genetic Analyzer (Thermo Fisher Scientific, Waltham, MA, USA). Sanger sequencing results from NB2 amplicons were analysed using the Standard Nucleotide—Basic Local Alignment Search Tool (BLAST) on the National Centre for Biotechnology Information (NCBI) website to confirm HPV-16 homology (see Supplemental Figure S2).

### 2.6. Statistical Methods

In order to examine the potential association between oral health and HPV-16 infection, HPV-16 DNA positivity results were analyzed against patient oral health status and periodontal disease status, as well as other recorded individual parameters (plaque, supra/sub-gingival calculus levels, gender, age, race, smoking and alcohol consumption status), using Fisher’s exact test. IBM SPSS v22 (IBM Corp, Armonk, NY, USA) was used as data analysis software. Fisher’s exact method was used to obtain *p*-values (exact significance, 1-sided) for variables with 2 × 2 contingency tables (gender, oral health status, periodontal status); for variables with contingency tables greater than 2 × 2 (plaque, supra/sub-gingival calculus levels, age, race, smoking and alcohol consumption status), Monte Carlo method (confidence level 99%, sample = 10,000) was used to obtain *p*-values (Monte Carlo significance, 2-sided). *p*-values less than 0.05 were considered significant. If the p value for a specific variable was significant, post-hoc testing was performed to determine *p*-values for each group within the variable. A significant *p*-value indicated that a specific sub-group within each variable was significantly associated with HPV-16.

## 3. Results

### 3.1. Patient Demographics

Participants (*n* = 223) were recruited from UQDS. Ten (4.5%) of these participants were confirmed to be HPV-16 DNA positive using NB2 endpoint PCR followed by Sanger sequencing confirmation.

Participants had a median age of 41 years and were distributed such that 53 participants (23.8%) were 18–30 years old, 106 (47.5%) subjects were 31–50 years old, 60 (26.9%) subjects were 51–70 years old, 4 (1.8%) were 71–90 years old. While more male 137 (61.4%) than female 86 (38.6%) participants were recruited, the age and gender differences between participants were not statistically significant. Demographically, 166 (78.3%) participants were identified as Caucasians, 26 (12.3%) Asians, 10 (4.7%) South Asians, 4 (1.9%) Aborigine or Pacific Islanders, 1 (0.5%) African, 4 (1.9%) Latino and 1 (0.5%) other race.

### 3.2. Lifestyle Related Risk Factors/Parameters

The prevalence of daily smoking amongst participants was 19.0%, which favorably compared with previous data from a similar UQDS population (22.9%) [34]. A significant proportion of participants were identified as former smokers (33.0%). Four out of the ten HPV-16 DNA positive participants did not disclose their smoking status. As such, any association between smoking and HPV-16 DNA infection could not be ascertained.

More than half (52.1%) of recruited participants were identified as regular consumers of alcohol, with 10.6% consuming more than one or two standard drinks per day. In total, 35 participants did not disclose their alcohol consumption patterns. A broad variation of alcohol consumption habits was noted amongst both HPV-16 DNA positive and HPV-16 negative participants. Neither frequency nor engagement in infrequent or frequent alcohol consumption were found to be associated with HPV-16 DNA prevalence (*p* = 0.147).

### 3.3. Oral Health Parameters

Almost all participants recruited demonstrated some level of plaque retention (98.6%). Supra (98.6%) and sub-gingival (90.0%) calculus presence was a common clinical finding amongst recruited participants. Neither the presence nor severity of these clinical parameters of gingival health was associated with HPV-16 DNA prevalence. Participants were also categorised based on their DMFT score. Of the study cohort, 35.9% (79) had a low measure of dental decay (past/present), whilst 42.7% (94) of participants experienced a moderate level of DMFT and 21.4% (47) of participants recorded a high score of DMFT. Nevertheless, DMFT score was not significantly associated with HPV-16 DNA infection (*p* = 0.259). While periodontal disease status was not significantly associated with the presence of HPV-16 DNA (*p* = 0.367), a large periodontal disease burden (40.5%) was noted within the recruited participants requiring further dental assistance.

Of the 10 HPV-positive participants, four patients presented with mild plaque build-up whilst six participants presented with moderate plaque build-up. Based on the aforementioned criteria, three participants presented with good to excellent oral hygiene whilst seven presented with poor to fair oral hygiene. Although the trends concur with our hypothesis, we did not find a statistically significant association between clinical oral health parameters and HPV-16 DNA prevalence status (Table 1).

## 4. Discussion

This is one of the first HPV studies to use oral health parameters examined and reported by dental professionals. In our study, the prevalence of HPV-16 DNA was found to be 4.5%, a rate comparable to previous studies by Gillison et al. (6.9%) and Antonsson et al. (2.3%) [4,35]. Men showed a lower HPV-16 infection rate (3.6%) than women (5.8%). Albeit not being statistically significant, our results showed a positive correlation between oral HPV-16 infection and poor oral hygiene. There was no statistically significant association found between HPV-16 DNA detection and socio-demographic or clinical oral health parameters.

A range of potential risk factors for oral HPV-16 infections, such as close contact with HPV-positive parents during early childhood [12], or maternal cervical/vaginal contact with a HPV-positive mother during birth [36] have been proposed. In addition, it is widely accepted that oral sexual activities are an established risk factor for HPV-16 infection [15,25,26,27,28,37,38,39]. In the current study, no social history and/or history of oral sex activities were collated. In a previous study, Bui et al. [15] observed an association between self-reported poor oral health and the risk of HPV-16 infection. An important differentiation factor between our study and the latter work is the distinction between clinically measured oral health parameters in our study as opposed to self-reported subjective oral health measures reported by Bui et al. The latter researchers also found self-reported oral health status to be an independent risk factor for oral HPV infection (OR = 1.55; 95% CI, 1.15–2.09). Similar trends of odds ratios were observed in our study between clinical oral hygiene status and HPV-16 infections (OR = 2.08; 95% CI, 0.52–8.27), though the wide confidence interval reflects the relatively low incidence of infection, and smaller sample size, in the present study.

It has been suggested by Tezal et al. that oral HPV infections may modify periodontal disease, and interact in a complex manner [28,36,40]. It has been proposed that instead of periodontal infections creating a suitable niche for oral HPV infections, it is also possible that oral HPV infection will exacerbate periodontal diseases [28,36]. Although gingivitis is an essential precursor for chronic periodontitis, not all gingivitis patients progress to chronic periodontitis, irrespective of the extent and duration of disease [28]. Multiple theories have been proposed for this two-way process of disease transformation, apart from the commonly accepted theory that periodontal patients elicit a different immune response due to their genetic predisposition [28,41]. One of these is that multi-organism synergy may play a role in the disease transformation process [28]. Virus-induced immunological responses may cause an imbalance between the host and pathogen, resulting in gingivitis–periodontitis transformation [28,41]. Additionally, HPV infection causes a cellular defect known as koilocytosis [39], whereby fragile defective cells may be more prone to potential periodontal breakdown [39,41]. Therefore, a model of the interaction between the two disease entities may be much more complex than hitherto envisioned and remains to be elucidated.

The major limitations in our study are the sample size. It is likely insufficient to detect a significant increase in HPV-16 infection associated with poor oral hygiene, as a minimum sample size of 150 per group would have been required for this purpose (power = 0.90, α = 0.05, anticipated detection rate in good oral hygiene group = 0.02). Another important consideration regarding the participants of this study is that the majority are patients who required treatment due to active oral disease within a university clinic setting. Consequently, the prevalence of poor oral hygiene and dental and periodontal infection in the study participants was not necessarily reflective of the general population in Australia.

## 5. Conclusions

In our pilot study population, the prevalence of HPV-16 DNA was found to be 4.5%. The results from this study highlight a positive correlation between oral HPV infection and poor hygiene, which has previously been documented to be an independent risk factor of HPV infection. Future studies with a larger study population (including healthy controls with premalignant disease as well as people with chronic inflammation) with active clinical oral health parameters may yield significant results. It has been advocated that general dental practitioners should advise patients about oral sexual behavior and HPV infection risk. However, for this practice to become part of routine dental consultative practices, many cultural and social hurdles need to be overcome [42] and clear evidence linking poor oral health to HPV infection needs to be presented.

## Figures and Tables

**Table 1:** Participant demographic and clinical parameters vs. human papillomavirus (HPV)-16 DNA status using oral rinse samples. DMFT, decayed, missing and filled teeth.

Variable	HPV-16 DNA Positive (% within Variable)	HPV-16 DNA Negative (% within Variable)	Total Number (% within Total Population)	*p*-Value
**Gender**				0.328
Male	5 (3.6)	132 (96.4)	137 (61.4)	
Female	5 (5.8)	81 (94.2)	86 (38.6)	
**Age**				0.130
18–30	1 (1.9)	52 (98.1)	53 (23.8)	
31–50	3 (2.8)	103 (97.2)	106 (47.5)	
51–70	6 (10.0)	54 (90.0)	60 (26.9)	
71–90	0 (0)	4 (100.00)	4 (1.8)	
**Race**				1.000
Caucasian	6 (3.6)	160 (96.4)	166 (78.3)	
Asian	0 (0)	26 (100.0)	26 (12.3)	
South Asian	0 (0)	10 (100.0)	10 (4.7)	
Aborigine/Pacific Islander	0 (0)	4 (100.0)	4 (1.9)	
African	0 (0)	1 (100.0)	1 (0.5)	
Latino	0 (0)	4 (100.0)	4 (1.9)	
Other	0 (0)	1 (100.0)	1 (0.5)	
**Smoking**				0.595
Never Smoker	2 (2.1)	95 (97.9)	97 (45.8)	
Former Smoker	4 (5.7)	66 (94.3)	70 (33.0)	
Current Daily Smoker <15	0 (0)	26 (100.00)	26 (12.3)	
Current Daily Smoker 15–24	0 (0)	12 (100.0)	12 (5.7)	
Current Daily Smoker 25–34	0 (0)	1 (100.0)	1 (0.5)	
Current Daily Smoker >35	0 (0)	1 (100.0)	1 (0.5)	
Current Non Daily Smoker	0 (0)	5 (100.0)	5 (2.4)	
**Alcohol consumption**				0.147
Non Drinker	1 (1.8)	54 (98.2)	55 (29.3)	
Infrequent Drinker	3 (8.6)	32 (91.4)	35 (18.6)	
Regular Drinker (<1/day for F, <2/day for M)	1 (1.3)	77 (98.7)	78 (41.5)	
Regular Drinker (>1/day for F, >2/day for M)	1 (5.0)	19 (95.0)	20 (10.6)	
**Plaque**				0.262
Nil	0 (0)	3 (100.0)	3 (1.4)	
Mild	4 (3.6)	108 (96.4)	112 (50.9)	
Moderate	6 (8.1)	68 (91.9)	74 (33.6)	
Severe	0 (0)	31 (100.0)	31 (14.1)	
**Calculus (Supra-gingival)**				0.182
Nil	0 (0)	3 (100.0)	3 (1.4)	
Mild	2 (1.9)	106 (98.1)	108 (49.1)	
Moderate	6 (7.3)	76 (92.7)	82 (37.3)	
Severe	2 (7.4)	25 (92.6)	27 (12.3)	
**Calculus (Sub-gingival)**				0.608
Nil	1 (4.5)	21 (95.5)	22 (10.0)	
Mild	3 (3.1)	95 (96.9)	98 (44.5)	
Moderate	4 (5.6)	68 (94.4)	72 (32.7)	
Severe	2 (7.1)	26 (92.9)	28 (12.7)	
**Oral Hygiene**				0.233
Good to Excellent	3 (2.9)	99 (97.1)	102 (46.4)	
Poor to Fair	7 (5.9)	111 (94.1)	118 (53.6)	
**DMFT**				0.259
0–9	6 (7.6)	73 (92.4)	79 (35.9)	
10–19	2 (2.1)	92 (97.9)	94 (42.7)	
20–28	2 (4.3)	45 (95.7)	47 (21.4)	
**Periodontal Status**				0.367
Non-Periodontal-Diseased	7 (5.3)	124 (94.7)	131 (59.5)	
Periodontal Diseased	3 (3.4)	86 (96.6)	89 (40.5)	

## Supplementary Materials

The following are available online at www.mdpi.com/2075-4418/07/1/11/s1.

## Abbreviations

HPV	human papillomavirus
OPC	oropharyngeal cancer
HNC	head and neck cancers
OC	oral cavity cancer
BOP	bleeding on probing
PSR	periodontal screening record
DMFT	decayed, missing and filled teeth index
