# Supplementary material for: A Pilot Study into the Association between Oral Health Status and Human Papillomavirus—16 Infection
Source: Diagnostics (Basel). 2017 Mar 1;7(1):11. doi: 10.3390/diagnostics7010011 (PMC5373020; doi:10.3390/diagnostics7010011)

**Table S1.** The participants’ demographic and lifestyle data.

| **Variable** | **Total Number (% within total population)** |
| --- | --- |
| **Gender** |  |
| Male | 137 (61.4) |
| Female | 86 (38.6) |
| **Age (years)** |  |
| 18–30 | 53 (23.8) |
| 31–50 | 106 (47.5) |
| 51–70 | 60 (26.9) |
| 71–90 | 4 (1.8) |
| **Race** |  |
| Caucasian | 166 (78.3) |
| Asian | 26 (12.3) |
| South Asian | 10 (4.7) |
| Aborigine/ Pacific Islander | 4 (1.9) |
| African | 1 (0.5) |
| Latino | 4 (1.9) |
| Other | 1 (0.5) |
| Not Available | 11 (not included in analysis) |
| **Smoking** |  |
| Never Smoker | 97 (45.8) |
| Former Smoker | 70 (33.0) |
| Current Daily Smoker <15 | 26 (12.3) |
| Current Daily Smoker 15–24 | 12 (5.7) |
| Current Daily Smoker 25–34 | 1 (0.5) |
| Current Daily Smoker >35 | 1 (0.5) |
| Current Non-Daily Smoker | 5 (2.4) |
| Not available | 11 (not included in analysis) |
| **Alcohol consumption** |  |
| Non-Drinker | 55 (29.3) |
| Infrequent Drinker | 35 (18.6) |
| Regular Drinker (<1/day for F, <2/day for M) | 78 (41.5) |
| Regular Drinker (>1/day for F, >2/day for M) | 20 (10.6) |
| Not available | 35 (not included in analysis) |

**Table S2**. The participants’ clinical data.

| **Variable** | **Number of participants (% within total population)** |
| --- | --- |
| **Plaque** |  |
| Nil | 3 (1.4) |
| Mild | 112 (50.9) |
| Moderate | 74 (33.6) |
| Severe | 31 (14.1) |
| Not available | 3 (not included in analysis) |
| **Calculus (Supra-gingival)** |  |
| Nil | 3 (1.4) |
| Mild | 108 (49.1) |
| Moderate | 82 (37.3) |
| Severe | 27 (12.3) |
| Not available | 3 (not included in analysis) |
| **Calculus (Sub-gingival)** |  |
| Nil | 22 (10.0) |
| Mild | 98 (44.5) |
| Moderate | 72 (32.7) |
| Severe | 28 (12.7) |
| Not available | 3 (not included in analysis) |
| **Oral Hygiene** |  |
| Good to Excellent | 102 (46.4) |
| Poor to Fair | 118 (53.6) |
| Not available | 3 (not included in analysis) |
| **DMFT** |  |
| 0–9 | 79 (35.9) |
| 10–19 | 94 (42.7) |
| 20–28 | 47 (21.4) |
| Not available | 3 (not included in analysis) |
| **Periodontal Status** |  |
| Non-Periodontal-Diseased | 131 (59.5) |
| Periodontal Diseased | 89 (40.5) |
| Not available | 3 (not included in analysis) |

**Lane 1**

**Lane 3**

**Lane 2**

**~140 bp**

**~250 bp**

**Lane 4**

**Lane 5**

**HPV 16 E7**

**Beta-globin**


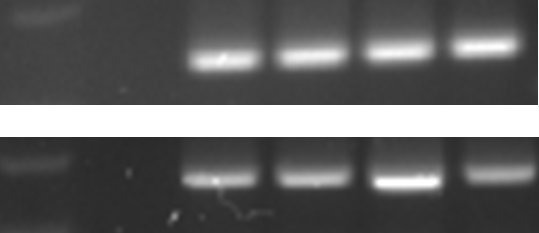


**Lane 6**

**Figure S1.** The detection of HPV-16 DNA in oropharyngeal cancer patients’ oral rinse samples (from Princess Alexandra Hospital, Brisbane, Australia) using NB-2 primers in a PCR. Representative gel image showing the detection of HPV-16 DNA in patient samples using HPV-16 NB2 primers (~140 bp) with beta-globin used as an internal control (~250 bp). Lane 1 represents DNA Ladder; Lane 2 represents non-template control; and Lanes 3–6 represent the presence of HPV-16 DNA in oropharyngeal cancer patient samples. The presence of HPV-16 DNA in patient samples was further confirmed by using Sanger sequencing.

**Figure S2.** BLAST Results

Oral rinse samples from HPV-16 positive UQ Dental School Samples (n = 10)

UQ121

GAACACACGGTAGACTTCGTACTTTGGAGACATGTTATGGGCACACTTGGAATTGTGTGCCCCATCTGTTCTCAGAAACCGTAATCTACCATGGCTGATCCTGCAGAACGCACAACCGAAGCGTAGAGTAAAA


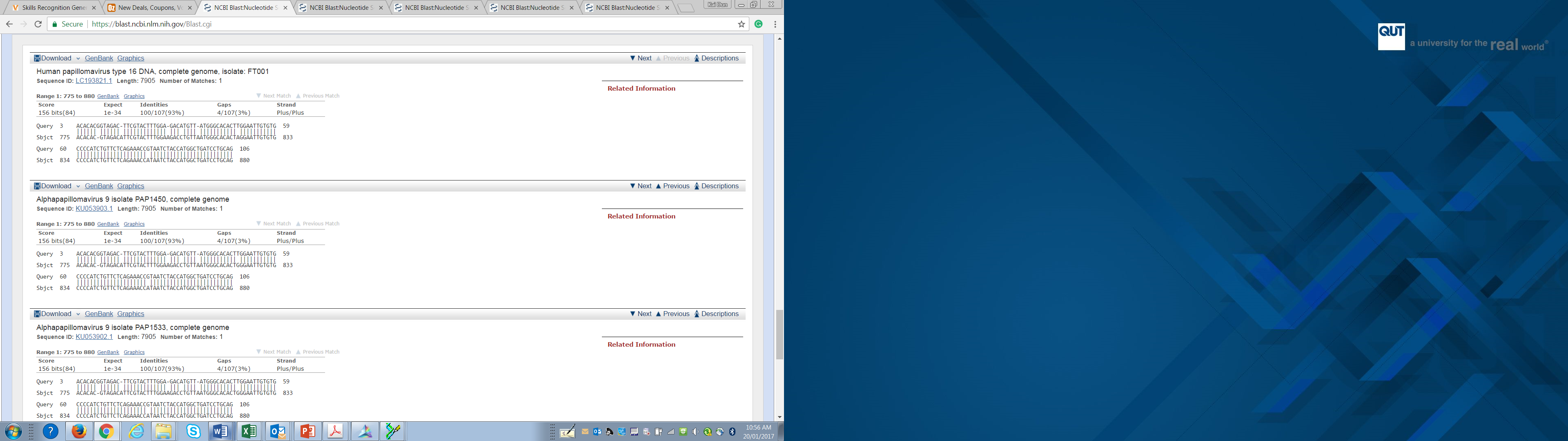


UQ138

GCTCATCGTAGACTTCGTACTTTGGAGACCTGTTATGGGCACACATAGGAATTGTGTGCCCCATCTGTTCTCAGAAACCATAATCTACCATGGCTGATCCTGCAGAC


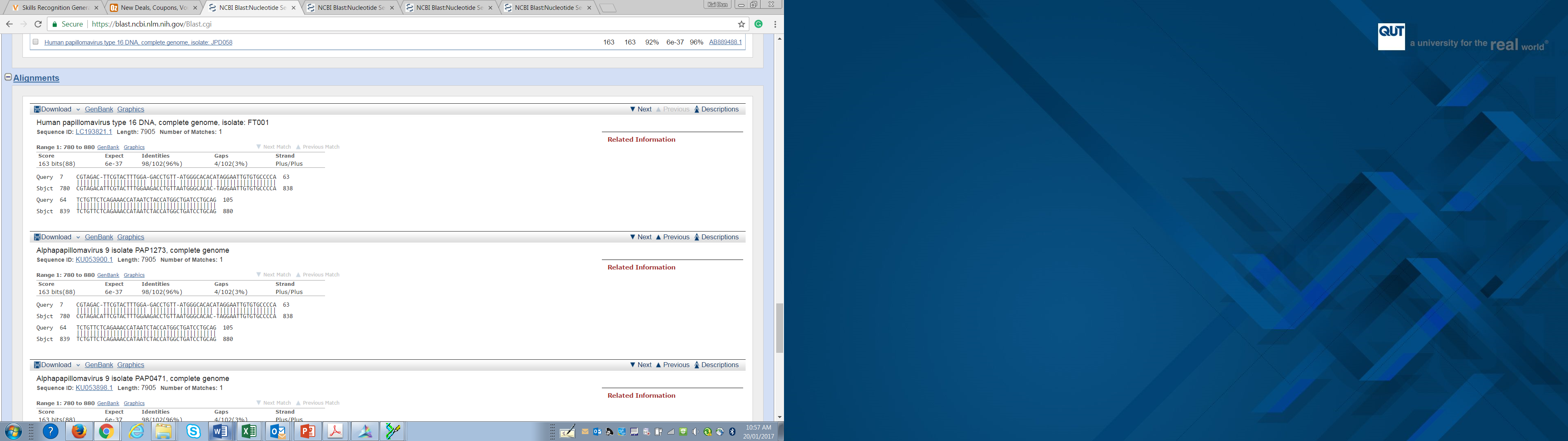


UQ220

ATCAACGTGCTCTTCGTACTTTGGAGACTGTTAATGGGCACACATAGGAATTGTGTGCCCCATCTGTTCTCAGAAACCATATTCTACCTTGGCTGAGCCTGCAGATTAATATACGCTATTGGAGCTGGAATTACCATGGCTGATCCTG


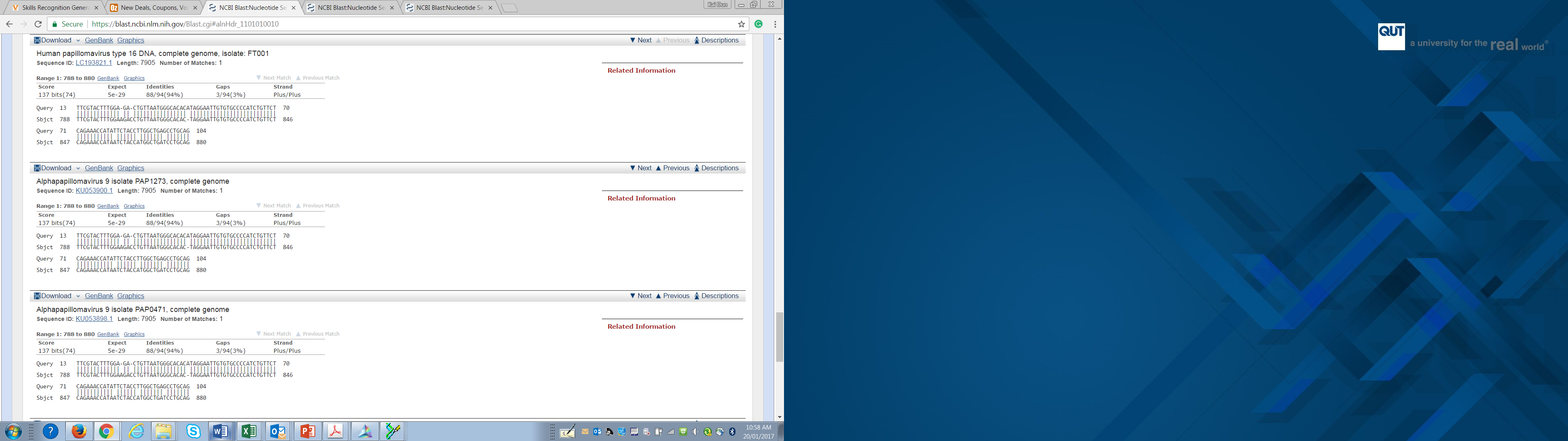


UQ236

GTACGAAGATGGGGCAACATTCCTAGTGTGCCCATTAACAGGTCTTCCAAGTACGAATGTCTACGTGTGTGCTTTGTACGCGCAACCGAAGCGTAGAGTCACACTAGGATGCTCTTAGCTGAGTGTCCTGCGGCCCGAAGCGTAGAGTCACACAG


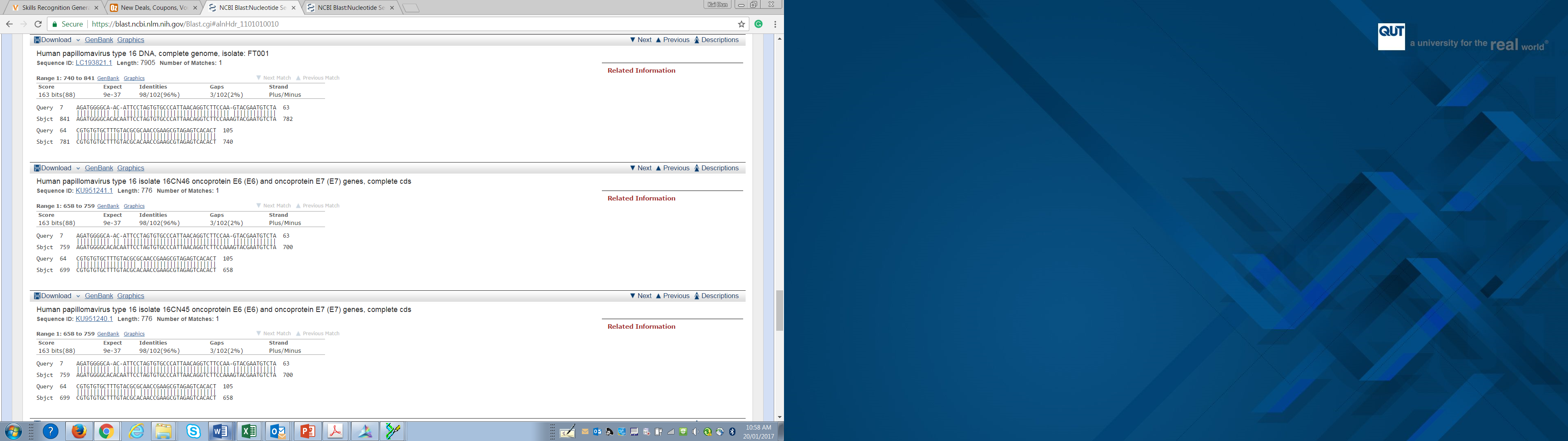


UQ237

GATTGACGATGGGCAACATTCCTAGTGTGCCCATTAACAGGTCTTCCAAAGTACGAATGTCTACGTGTGTGCTTTGTACGCACAACCGAAGCGTAAAGTCACACTCGA


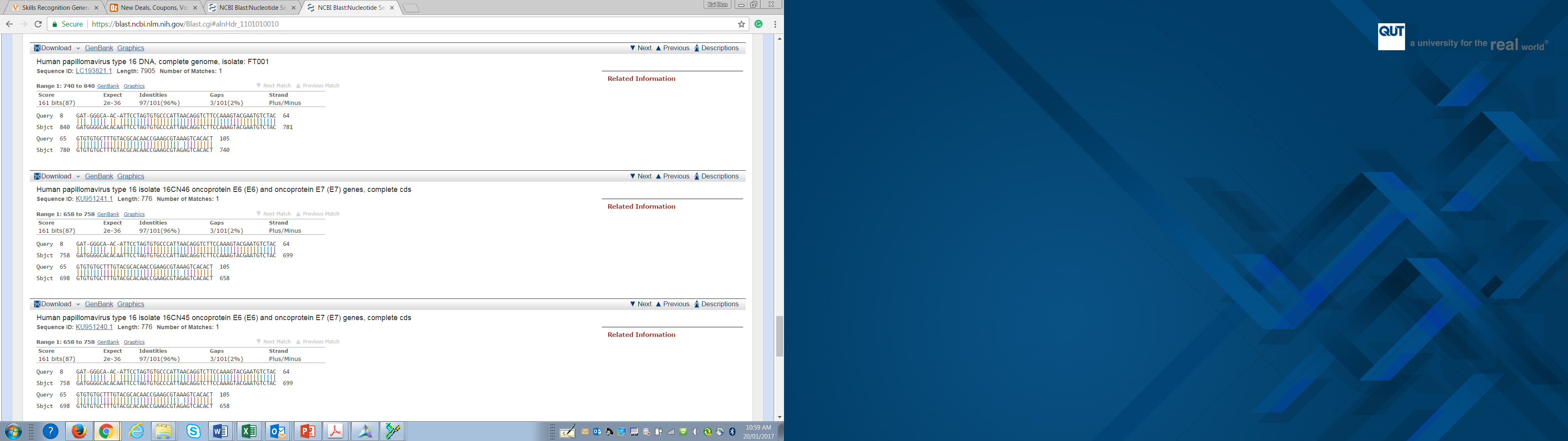


UQ256

AGTAGCCGGAACATATTAGGGCAACAATTCCTAGTGTGCCCATTAACAGGTCTTCCAAAGTACGAATGTCTACGTGTGTGCTTTGTACGCACAACCGAAGCGTAGAGTCACACTCGA


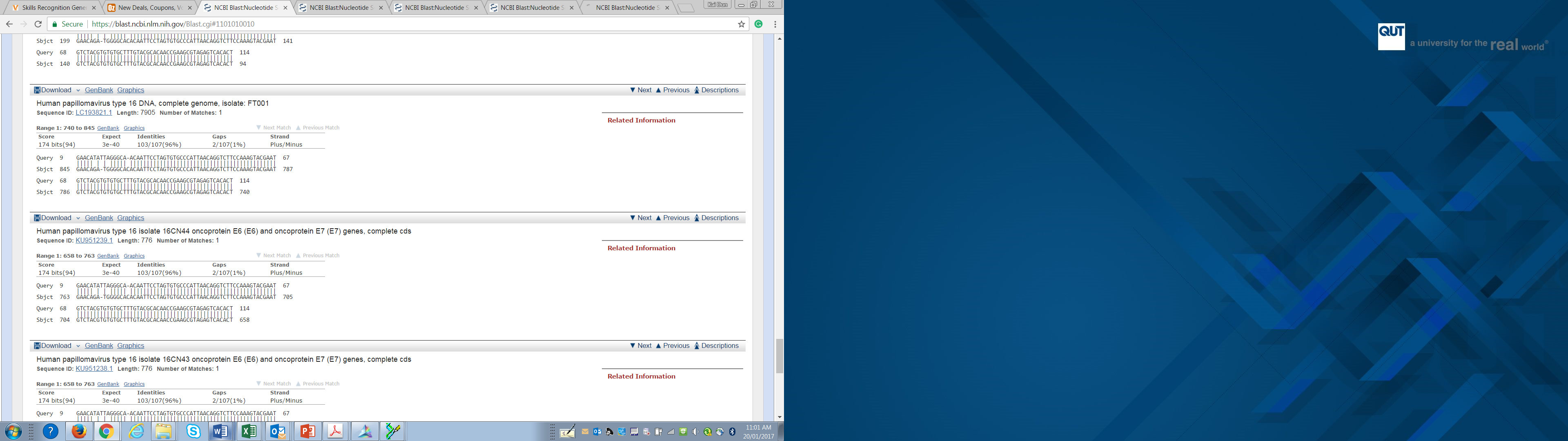


UQ1338

ACCCCGTAGACTTCGTACTTTGGAGACCTGTTATGGGCACACTAGGAATTGTGTGCCCCATCTGTTCTCAGAAACCATAATCTACCATGGCTGATCCTGCAG


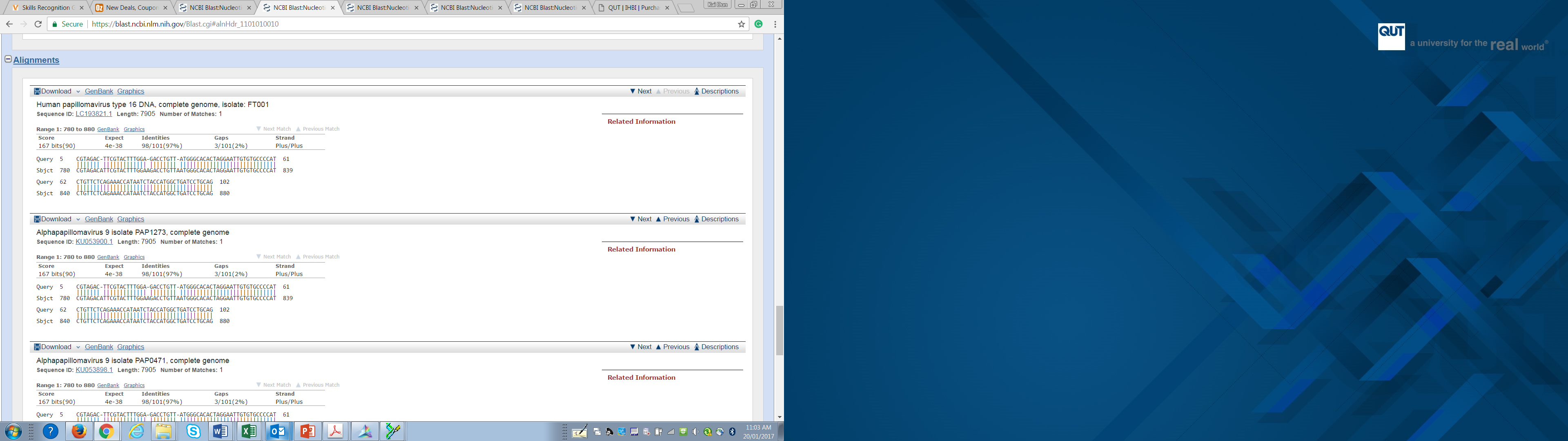


UQ1359

GCAAACTTAGACTTCGTACTTTGGAGACCTGTTAATGGGCACACTAGGAATTGTGTGCCCCATCTGTTCTCAGAAACCATAATCTACCATGGCTGATCCTGCAG


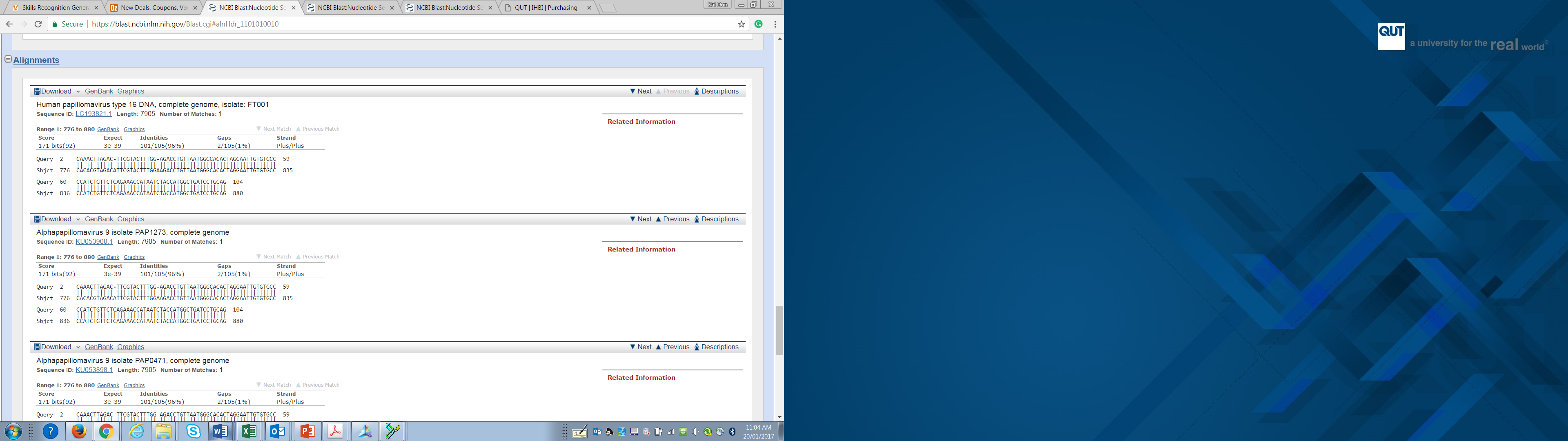


UQ1367

ACACCTTAGACTTCGTACTTTGGAGACCTGTTAATGGGCACACTAGGAATTGTGTGCCCCATCTGTTCTCAGAAACCATAATCTACCATGGCTGATCCTGCAG


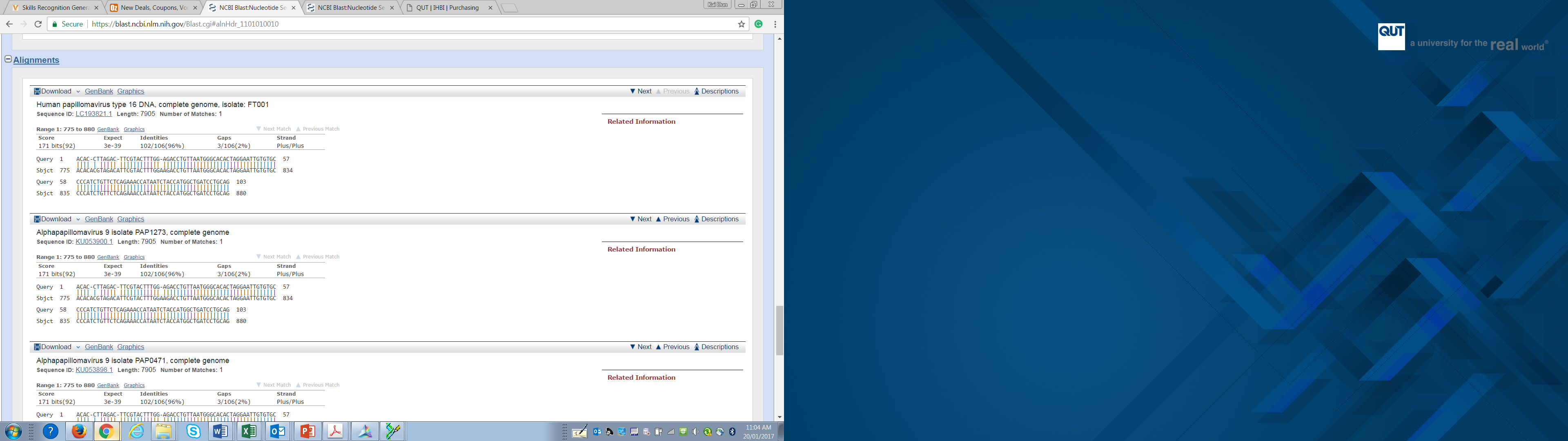


UQ1368

GCAAACGCTACACTTCGTACTTTGGAGACCTGTTATGGGCACACTAGGAATTGTGTGCCCCATCTGTTCTCAGAAACCATAATCTACCATGGCTGATCCTGCAG


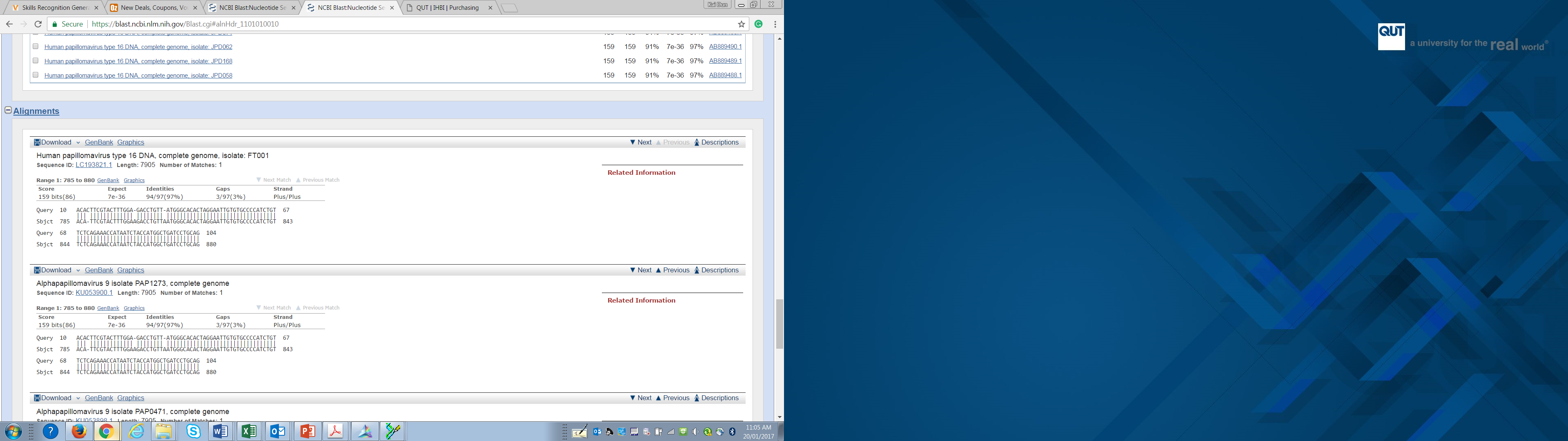


Oral rinse samples from HPV-16 positive oropharyngeal patients (Supplementary Figure S1)

Lane 3

GGCTTTGCCACACGTAGACATTCGTACTTTGGAAGACCTGTTAATGGGCACACTGGGAATTGTGTGCCCCATCTGTTCTCAGAAACCATAATCTACCATGGCTGATCCTGCAG


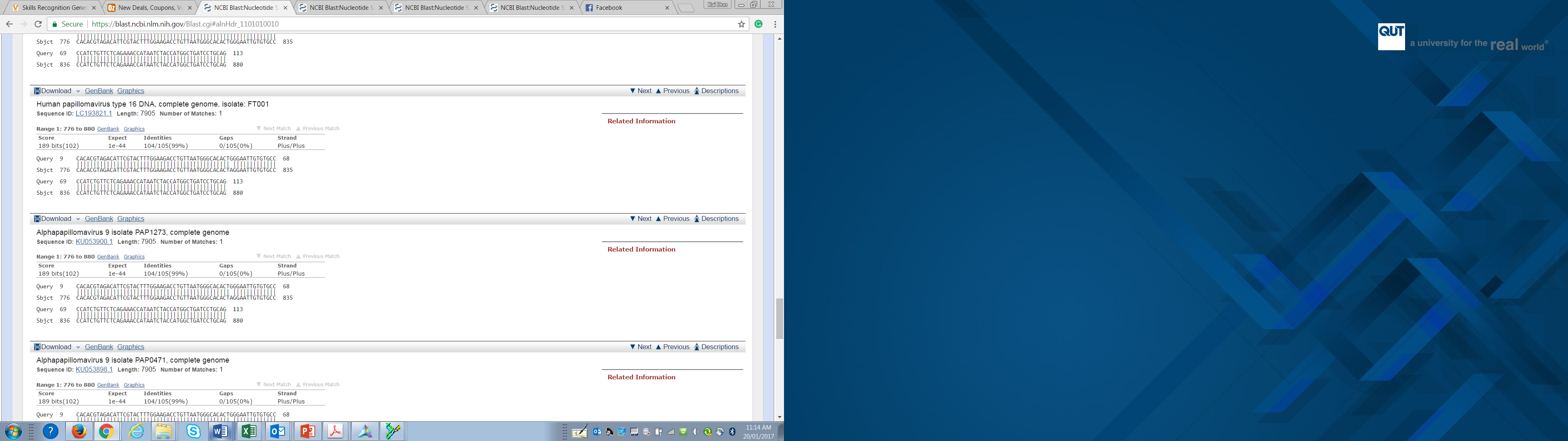


Lane 4

AGTCAGCACACGTAGACATTCGTACTTTGGAGACCTGTTAATGGGCACACTAGGAATTGTGTGCCCCATCTGTTCCCAGAAACCATAATCTACCATGGCTGATCCTGCAG


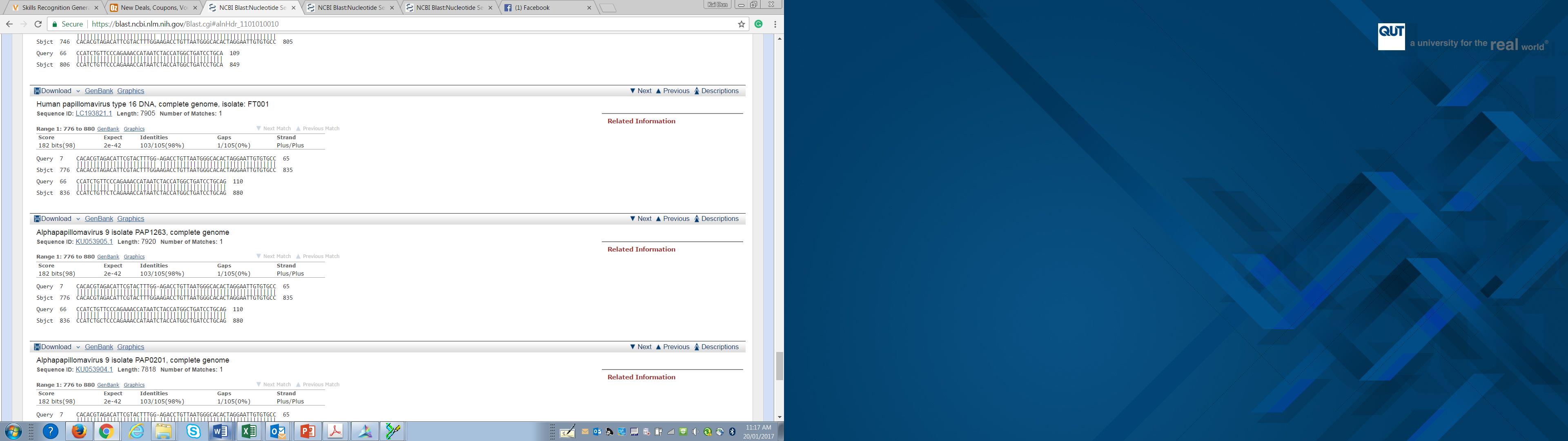


Lane 5

TGGGTCATGCCACACGTAGACATTCGTACTTTGGAGACCTGTTAATGGGCACACATAGGAATTGTGTGCCCCATCTGTTCTCAGAAACCATAATCTACCATGGCTGATCCTGCAGA


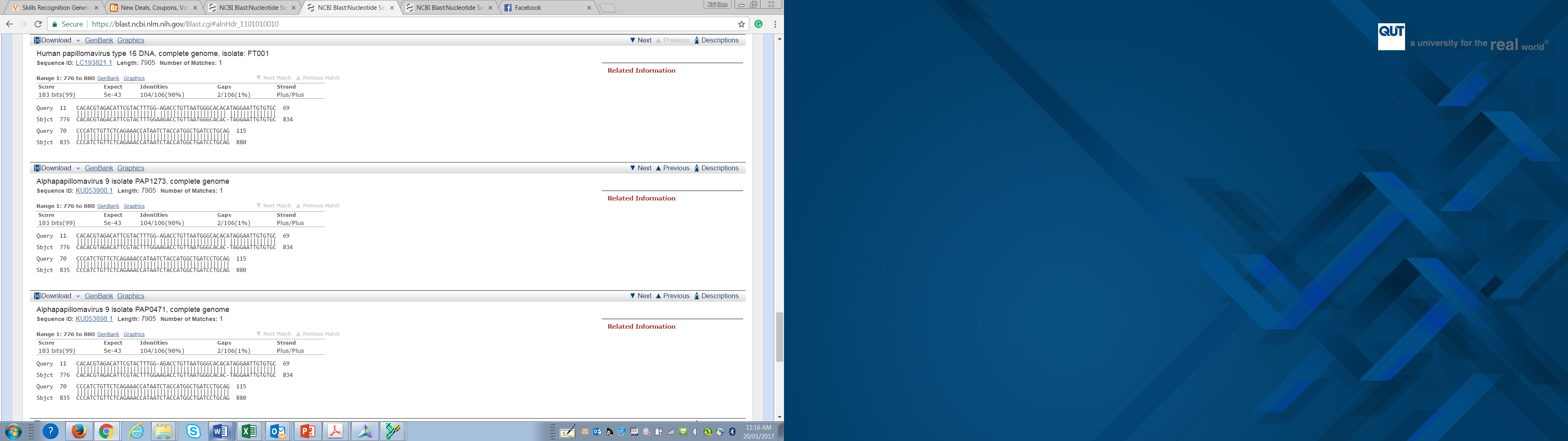


Lane 6

GTGGGCAGCACACGTAGACATTCGTACTTTGGAGACCTGTTAATGGGCACACATAGGAATTGTGTGCCCCATCTGTTCTCAGAAACCATAATCTACCATGGCTGATCCTGCAGA


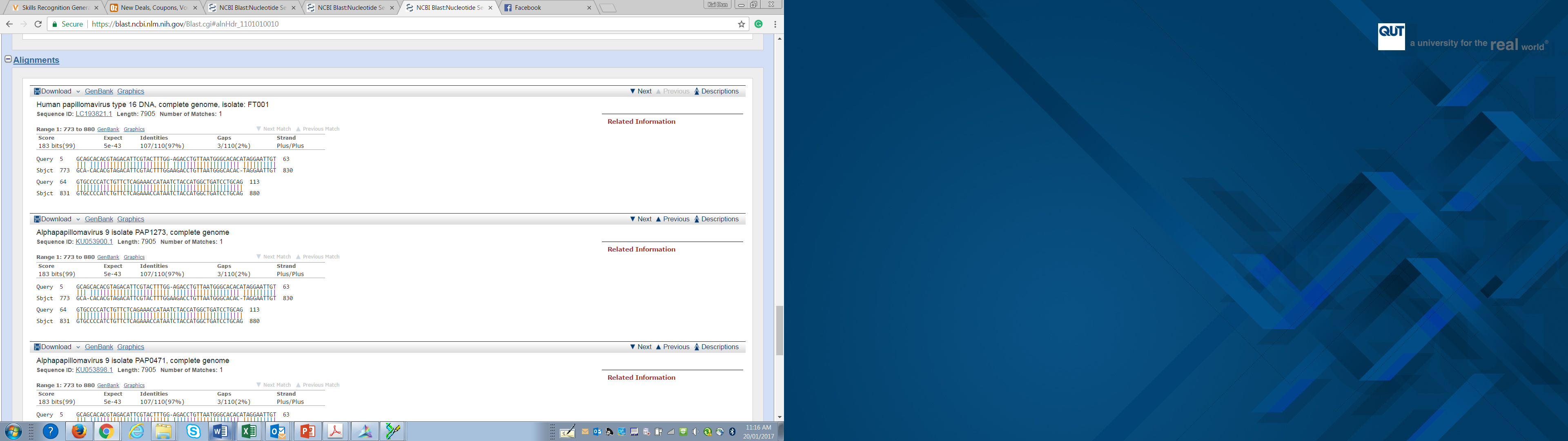

Supplement: Supplementary file 1 [file diagnostics-07-00011-s001.docx]
